# Blue cone monochromacy: Causative mutations and associated phenotypes

**Published:** 2009-05-01

**Authors:** Jessica C. Gardner, Michel Michaelides, Graham E. Holder, Naheed Kanuga, Tom R. Webb, John D. Mollon, Anthony T. Moore, Alison J. Hardcastle

**Affiliations:** 1 Institute of Ophthalmology, University College London, London, UK; 2 Moorfields Eye Hospital, London, UK; 3 Department of Experimental Psychology, University of Cambridge, Cambridge, UK

## Abstract

**Purpose:**

To perform a phenotypic assessment of members of three British families with blue cone monochromatism (BCM), and to determine the underlying molecular genetic basis of disease.

**Methods:**

Affected members of three British families with BCM were examined clinically and underwent detailed electrophysiological and psychophysical testing. Blood samples were taken for DNA extraction. Molecular analysis involved the amplification of the coding regions of the long (L) and medium (M) wave cone opsin genes and the upstream locus control region (LCR) by polymerase chain reaction (PCR). Gene products were directly sequenced and analyzed.

**Results:**

In all three families, genetic analysis identified that the underlying cause of BCM involved an unequal crossover within the opsin gene array, with an inactivating mutation. Family 1 had a single 5′-L–M-3′ hybrid gene, with an inactivating Cys203Arg (C203R) mutation. Family 3 had an array composed of a C203R inactivated 5′-L–M-3′ hybrid gene followed by a second inactive gene. Families 1 and 3 had typical clinical, electrophysiological, and psychophysical findings consistent with stationary BCM. A novel mutation was detected in Family 2 that had a single hybrid gene lacking exon 2. This family presented clinical and psychophysical evidence of a slowly progressive phenotype.

**Conclusions:**

Two of the BCM-causing family genotypes identified in this study comprised different hybrid genes, each of which contained the commonly described C203R inactivating mutation. The genotype in the family with evidence of a slowly progressive phenotype represents a novel BCM mutation. The deleted exon 2 in this family is not predicted to result in a shift in the reading frame, therefore we hypothesize that an abnormal opsin protein product may accumulate and lead to cone cell loss over time. This is the first report of slow progression associated with this class of mutation in the L or M opsin genes in BCM.

## Introduction

Blue cone (S cone) monochromatism (BCM) is a rare X-linked congenital stationary cone dysfunction syndrome, affecting approximately 1 in 100,000 individuals. Affected males with BCM have no functional long wavelength sensitive (L) or medium wavelength sensitive (M) cones in the retina. Color discrimination is severely impaired from birth, and vision is derived from the remaining preserved S cones and rod photoreceptors [1,2]. BCM typically presents with reduced visual acuity (6/24 to 6/60), pendular nystagmus, photophobia, and patients often have myopia. The rod-specific and maximal electroretinogram (ERG) usually show no definite abnormality, whereas the 30Hz cone ERG cannot be detected. Single flash photopic ERG is often recordable, albeit small and late, and the S cone ERG is well preserved [3]. In addition to electrophysiological assessment, psychophysical testing can be readily used to identify the characteristic retained tritan discrimination in patients with BCM. These tests include the Farnsworth-Munsell 100-Hue test, Berson color plates, Hardy, Rand, and Rittler (HRR) plates, and the standard and enlarged Mollon-Reffin (MR) Minimal test [4-7].

S (blue), M (green), and L (red) visual pigments confer the different cone absorption spectra. The L and M genes in the genomic array on Xq28 are situated in a head to tail tandem arrangement with a single L opsin gene in a 5′ position followed by one or more M opsin genes [1,8]. The number of M genes is polymorphic: Approximately 25% of male caucasians have a single M gene, while 50% have two M genes and the remainder have 3 or more genes [1,8,9]. Expression of the L and M genes is regulated by the Locus Control Region (LCR), a conserved sequence situated roughly 3.5 kb upstream of the L gene. The LCR plays a critical role in ensuring that only one opsin gene in the array is expressed in a single cone photoreceptor [10]. It has also been demonstrated that only the first two genes of the array are expressed in the retina [11].

The L and M cone opsins are encoded by six-exon genes, which are highly homologous and share 96% amino acid identity, while their homology to S cone opsin and rod opsin is approximately 40% [1,8]. Seven of the 15 amino acids that differ between the L and M opsins account for the major differences in spectral tuning between the two photopigments. Amino acids at positions 180, 277, and 285 have a major effect on spectral tuning, while amino acids 116, 230, 233, and 309 confer a minor effect [12,13]. The majority of these residues are encoded by exons 3 and 5 of the L and M opsin genes [12,13].

The molecular genetic mechanism underlying BCM was identified as L and M opsin gene array mutations, resulting in nonfunctional photopigments and thus inactive L and M cones [9,14]. The mutations identified in the L and M opsin gene array fall into three classes. In the first class (roughly 40% of cases), a normal L and M opsin gene array is inactivated by a deletion in the LCR [15-17]. The second class of mutations (approximately 60% of cases) is due to a two-step mutation mechanism of nonhomologous recombination and point mutation. Nonhomologous recombination between the L and M opsin genes reduces the number of genes in the opsin array to one. This step is followed by a single nucleotide sequence alteration (point mutation), which inactivates the residual gene. To date, three inactivating opsin mutations (C203R, R247X, and P307L) have been reported with C203R being described most frequently [7,9,15]. The most common BCM genotype in this class consists of a single inactivated L–M hybrid gene with a C203R mutation. A third class of mutation has been reported in a Danish BCM family where a single opsin array gene (L) was found to have a deletion of an entire exon (exon 4) [18].

In this study we describe a molecular genetic analysis of the L and M opsin array in three British families with BCM and their associated ocular phenotypes.

## Methods

### Patients

This study was approved by the Moorfields Eye Hospital Ethics Committee. Seven affected males (age range 5 years–76 years), 2 asymptomatic obligate carrier females and one unaffected female from three British families with BCM were examined and underwent detailed psychophysical and electrophysiological assessment. After informed consent was obtained, blood samples were taken. Genomic DNA was isolated from whole blood using an extraction kit (GE Healthcare Ltd, Buckinghamshire, UK) and molecular genetic analysis was performed.

### Clinical assessment

**Figure 1:**
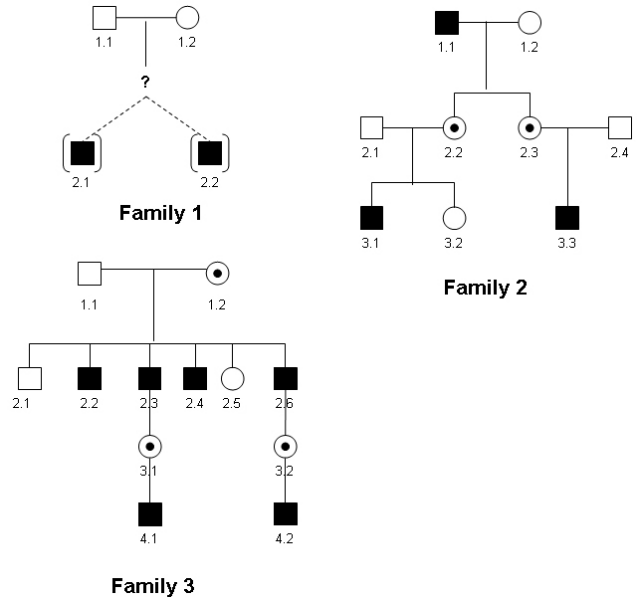
BCM family pedigrees. Affected males are represented by shaded boxes. Obligate female carriers are represented by circles with a central black dot. In Family 1 the affected twins were adopted with no known family history of BCM (represented by “?” symbol). The pedigrees of Families 2 and 3 show an X-linked pattern of inheritance.

The pedigrees of the three families studied are shown in Figure 1. Families 2 and 3 show an inheritance pattern consistent with X-linked inheritance. In Family 1 the affected twins were adopted with no known family history. A medical and ophthalmic history was taken, and a full ophthalmological examination was performed in all examined participants. All affected individuals were given a full-field electroretinogram (ERG). ERG procedures conformed to the International Society for Clinical Electrophysiology of Vision standard [19], with the children undergoing a modified protocol using skin electrodes. S cone ERGs were also recorded in Families 1 and 2 using a previously described protocol [20].

Color vision testing included the use of the Ishihara pseudoisochromatic plates, HRR plates (American Optical Company, New York, NY), Farnsworth-Munsell (FM) 100-hue test, Farnsworth D-15 and enlarged Farnsworth D-15 (PV-16), the standard and enlarged MR test [6], a computerized color vision test [21,22], and anomaloscopy. The FM 100-hue, Farnsworth D-15/PV-16 and the MR tests were all performed under CIE Standard Illuminant C from a MacBeth Easel lamp.

### Molecular genetic analysis

Amplification of the LCR and exons 1 to 6 of the L and M opsin genes was undertaken in an affected male from Families 1 to 3 (Figure 1). Subsequently further male family members and carrier females were screened to determine segregation. Population control male DNA samples were also used, in which we had previously sequenced the L and M opsin genes. Since the L and M gene sequences (exonic and intronic) are 98% identical, it was possible to amplify the exons of both genes using one primer pair. PCR reactions were performed to amplify a fragment containing the LCR and to coamplify each of the six exons of both the L and M opsin genes for each subject essentially as previously described [7] with modifications (primers and conditions available on request). PCR reactions (25 µl) contained the following: 3 µl of 200 ng genomic DNA, 12.5 µl 2X Mastermix (AB-0575, Abgene, Thermo Fisher Scientific, Leicestershire, UK), 3 µl of 2 mM forward and 3 µl of 2 mM reverse primer and 3.5 µl dH_2_O. Cycling conditions were as follows: 95 °C for 4 min followed by 35 cycles of 95 °C for 30 s, annealing temperature (Ta) °C for 30 s, 72 °C for 30 s, followed by 1 cycle of 72 °C for 4 min. PCR products were analyzed by agarose gel electrophoresis. All amplified products were then purified enzymatically. In dH_2_O, 1 µl ExoSAP-IT (GE Healthcare Ltd, Buckinghamshire, UK) was added to an aliquot of PCR product (1–6 µl containing approximately 500 ng) to create a volume of 26 µl. The samples were incubated at 37 °C for 15 min and heat inactivated at 80 °C for 15 min. An aliquot (13 µl) of purified PCR product was then bidirectionally sequenced with 1 µl forward or reverse primer (2 µM stock), 1 µl BigDye (version 3.1) terminator cycle sequencing chemistry, and 5 µl buffer (Applied Biosystems, Warrington, UK) following the manufacturers' protocols. The product was analyzed on an ABI 3730 Genetic Analyzer (Applied Biosystems, Applera, UK). The sequence was examined for alterations with Lasergene DNA Star software (DNA Star Inc., Madison, WI).

Coamplification of L and M exons resulted in a mixed population of amplified fragments, which were cosequenced. Differentiation between L and M exons was achieved by the identification of known sequence differences in the resultant electropherograms, conferring different opsin spectral properties, present in exons 2 to 5. Coding sequence of L and M exons 1 and 6 were identical and could not be differentiated on an electropherogram; however, sequence upstream of the start codon of exon 1 contained nucleotide differences which facilitated identification of both M and L exon 1.

To specifically amplify either L exon 2 or M exon 2, we designed L and M gene-specific exonic primer pairs (Table 1). PCR and sequencing reactions were performed as described (see Table 1), and exon 2 gene specific products were successfully amplified and sequenced in control participants.

**Table 1:** PCR primers used to further analyze the opsin array in Family 2.

**L or M opsin specific exon 2 PCR primers**	**5′-3′ Sequence**	**Product size**	**Annealing temperature (Ta) °C**
LEx2F	ctggatgatctttgtggtcac	192 base pairs (bp)	59
LEx2R	cccagcacgaagtagccag		
MEx2F	ctggatgatctttgtggtcat	192 bp	56
MEx2R	cccagcacgaagtagccat		
**Long Range PCR primers**	**5′- 3′ Sequence**	**Product size**	**Ta °C**
LRF	ggctgcactgggggccac	~7–8 kilobases	70
LRR	aagcaaagcttcccactgtcctgcttagac		
**Internal opsin sequencing primers**	**5′- 3′ Sequence**		
Mint1F	tttctcacagctctggaggc		
Mint2R	agggagacaggcctaca		

Long wave sensitive (L) and Medium wave sensitive (M) opsin specific primer pairs were designed to independently amplify L or M exon 2 sequences. Primer pair LEx2F and LEx2R (Long wave sensitive Exon 2 Forward and Long wave sensitive Exon 2 Reverse) was used to amplify L exon 2 sequence only while primer pair MEx2F and MEx2R was used to amplify M exon 2 sequence only. The Long Range PCR primers (Long Range Forward [LRF] and Long Range Reverse [LRR]) were designed to co-amplify L and M gene sequence spanning the suspected hybrid gene deletion of exon 2 in Family 2. Internal opsin sequencing primers M gene intron 1 Forward (Mint1F) and M gene intron 2 Reverse (Mint2R) were designed to sequence the 7.5 kb PCR product amplified by the Long Range PCR primer pair.

Long-range PCR from L exon 1 to M exon 3 in Family 2 was performed as described in this section using AB-0793 PCR Master Mix (Abgene) and an intronic coamplifying L–M primer pair (Table 1) with a 6 min extension time and an annealing temperature of 70 °C. The PCR fragment was then directly sequenced with internal primers (Table 1).

## Results

### Family 1 phenotype

Since birth, the 11-year-old twin brothers of Family 1 (family members 2.1 and 2.2, Figure 1) were noted to have nystagmus, but this was found to have gradually decreased over time. The brothers complained of minimal photophobia and reduced color vision, and were not aware of any difficulty with night vision. One had epilepsy. They were adopted, and no family history was known. They were both myopic (−5.0 DS) with best corrected visual acuities of 6/24 in both eyes. On examination they had mild horizontal pendular nystagmus, normal fundi, and clear media.

Rod-specific ERGs were normal and 30 Hz cone flicker ERGs were undetectable in both brothers. In both brothers, single flash photopic ERGs demonstrated residual cone activity with marked delay; this was likely to be related to S cone mechanisms, owing to the good responses recorded to S-cone specific stimulation (S cone ERGs). Psychophysical testing of the twins with the standard and enlarged MR test and HRR plates revealed good discrimination along the tritan axis and minimal residual deutan and protan discrimination. They displayed a protan ordering of the PV-16, as has been previously described in BCM [23]. On computerized testing their color discrimination ellipses were oriented along the angle that one would expect of someone making color discriminations based on a comparison of quantum catches in the rods and S-cones.

### Family 1 genotype

**Figure 2:**
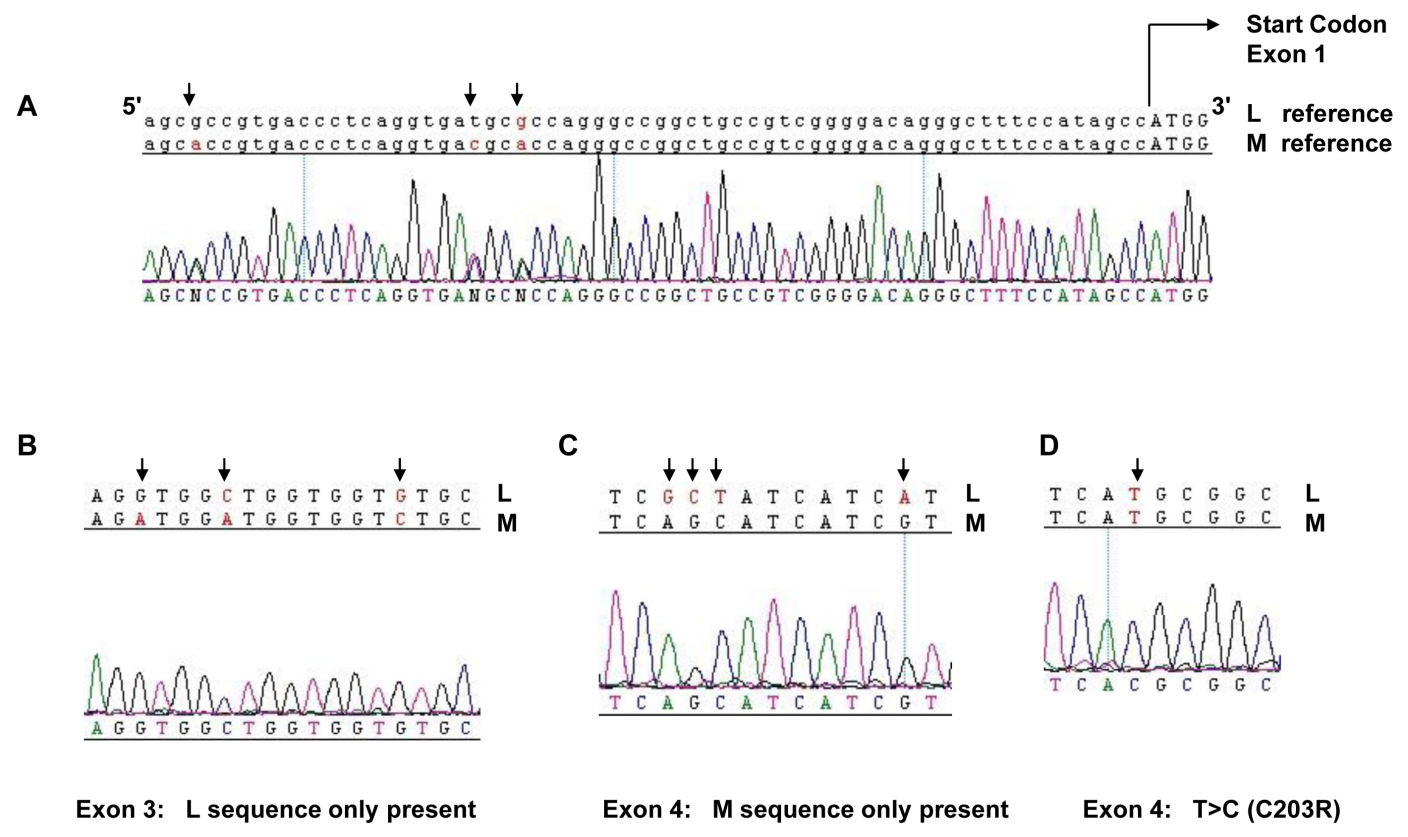
Sequence analysis of L and M opsin genes in Family 1. Sequence from an affected male (2.1) was generated with primers designed to coamplify both L and M genes. Reference sequences of the L and M genes are shown on top of the patient electropherograms. Nucleotides marked with an arrow indicate known differences between the L and M genes or a patient mutation. **A** shows electropherogram sequence in the region of exon 1 5′ to the start codon (indicated by capital letters). Note the double peaks (denoted N) at positions of sequence variation between L and M genes (arrows), indicating the presence of both L and M exon 1. **B** shows sequence from Exon 3. Single peaks at positions of known nucleotide variation (arrows) between the L and M genes indicate only L opsin exon 3 is present. **C** shows an electropherogram from Exon 4 which indicates M exon 4 sequence only is present. **D** shows a section of Exon 4 in which a T>C nucleotide substitution (arrow) is present that results in a C203R missense mutation.

**Figure 3:**
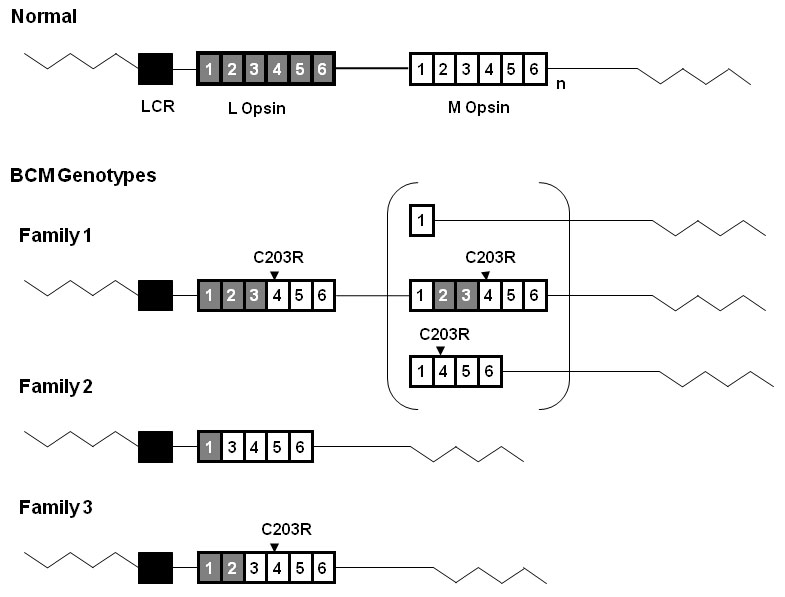
Diagram representing BCM genotypes of the 3 British families. The wild type L-M opsin gene array is shown at the top of the figure. Grey boxes represent L opsin exons, and white boxes represent M opsin exons. Subscript n represents one or more M opsin genes. The black box represents the Locus Control Region (LCR). The LCR was present without mutation in all three families. The C203R point mutations detected in Family 1 and Family 3 are shown above the corresponding exons. Family 1 has an inactive hybrid gene followed by a second gene in the array. Three possible structures of this second inactive gene are shown in the bracket. Family 2 has a single nonfunctional hybrid gene lacking exon 2. Family 3 has a single inactive hybrid gene.

Sequence analysis in family member 2.1 revealed no deletions or sequence variants of the LCR. Sequence representing exons 1, 2, and 3 of the L gene was identified, while exons 4 and 5 of the L gene were absent (Figure 2). Exons 1, 4, and 5 of the M gene were present, but M exons 2 and 3 were absent (Figure 2). The first gene in the array is therefore a 5′-L–M-3′ hybrid gene comprising L exons 1, 2, and 3 joined to M exons 4, 5, and 6 (Figure 3). Alignment of the hybrid gene with wild-type M and L sequence showed that this opsin gene carries a T>C nucleotide substitution in exon 4, encoding the Cys203Arg missense mutation (C203R) which is known to disrupt the folding of cone opsin (Figure 2D). Individual 2.2 was also found to have the same genotype.

Although the coding region sequences of exons 1 and 6 were identical in the L and M genes, nucleotide variation between the L and M sequences in the noncoding upstream region of exon 1 was detected, indicating that both L and M copies of exon 1 were present in affected members of the family (Figure 2A). This suggests that there is a second gene in the array with an M exon 1. Possible structures of this second gene are shown in Figure 3. All possible second genes were predicted to be inactive. The C203R mutation will be present in all genes in the array with an exon 4, as there is no wild-type exon 4 sequence (Figures 2C,D). In summary, BCM in Family 1 is due to a two-step mutational mechanism in which non homologous recombination has been followed by an inactivating point mutation. In this case, step one resulted in the formation of a 5′-L/M-3′ hybrid gene and step two resulted in inactivation of the hybrid by a missense C203R mutation in M exon 4. Although a second gene may be present in the array, this would also be non-functional owing either to the presence of the C203R missense mutation in M exon 4 or to a deletion.

### Family 2 phenotype

The detailed phenotype of this extended family has been previously reported [7]. In brief, both family member 3.1, age 12, and family member 3.3, age 7, had typical clinical features of BCM, with normal fundi. On electrophysiological testing, both had absent cone responses but normal rod responses. Detailed psychophysical testing revealed reasonable discrimination only along the tritan axis in both participants.

Their 60-year-old affected grandfather (1.1) complained that his vision had continued to slowly deteriorate throughout life. He had evidence of mild bilateral macular retinal pigment epithelial changes. Cone ERG responses were absent, but rod responses were normal; he had no residual color vision.

Since 3.1 and 3.3 both had good tritan color discrimination and their grandfather had no color discrimination, it would appear that their condition is not stationary. Both 3.1 and 3.3 had a typical BCM phenotype, whereas their grandfather behaved as a rod monochromat, presumably as a result of S cone loss. The lack of color vision seen in the grandfather is highly unlikely to be due to lenticular changes since his lenses were found to be clear.

### Family 2 Genotype

**Figure 4:**
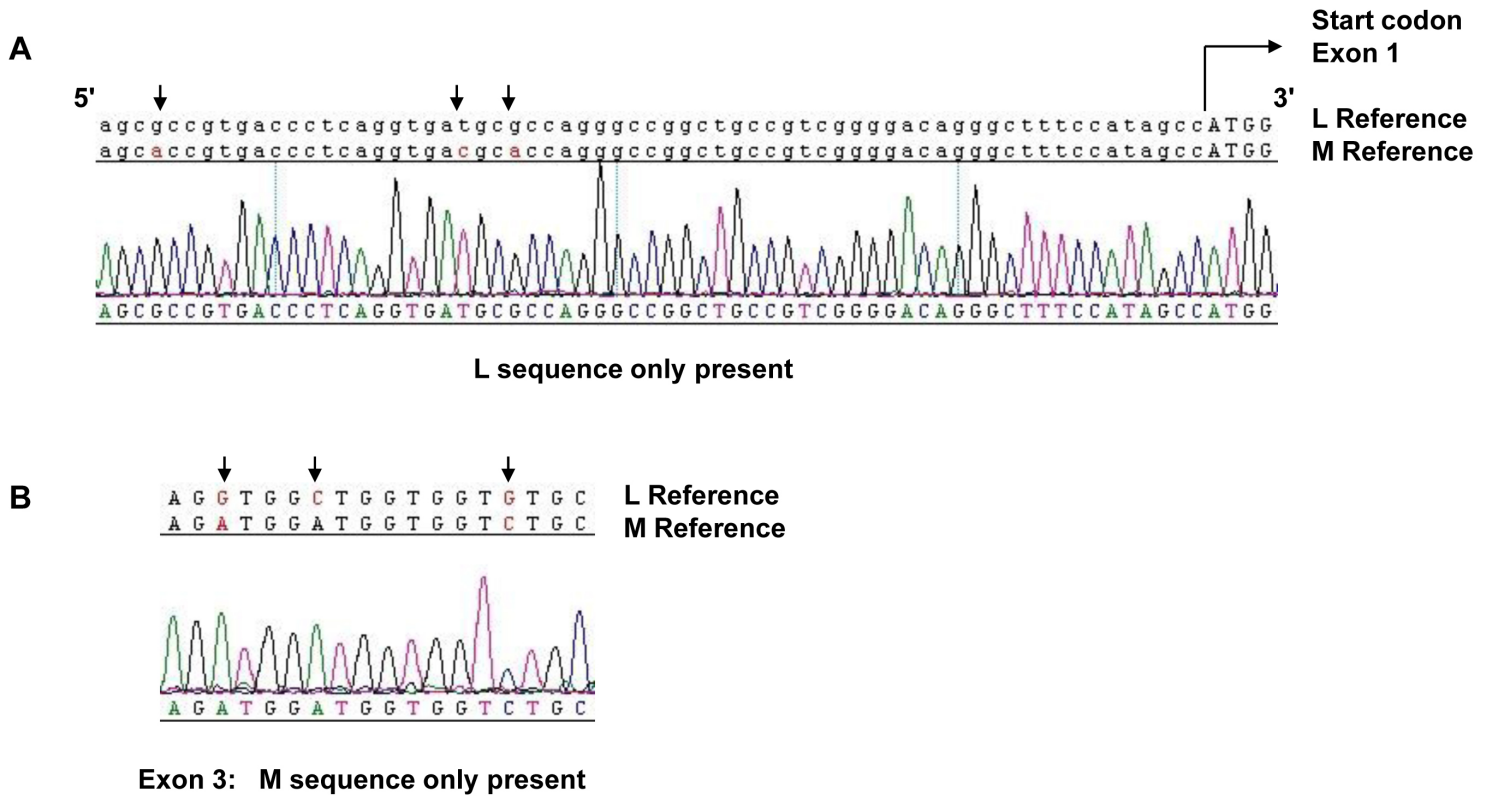
Sequence analysis of L and M opsin genes in Family 2. Reference sequences of the L and M genes are shown on top of the electropherograms, which were generated from an affected male (individual 1.1) in Family 2. Nucleotides marked with an arrow indicate known differences between the L and M genes. **A** shows opsin sequence beginning 5′ to the start codon (capital letters) of exon 1. Note the presence of single sequence peaks at positions of known nucleotide variation between L and M gene sequences (arrows), indicating the absence of M exon 1. **B** shows Exon 3 sequence with single peaks at sites of known variation between L and M opsin gene sequences (arrows) representing the presence of M exon 3 nucleotides and absence of L exon 3 nucleotides. Similarly, absence of an L sequence was noted for exons 4 and 5 (data not shown).

**Figure 5:**
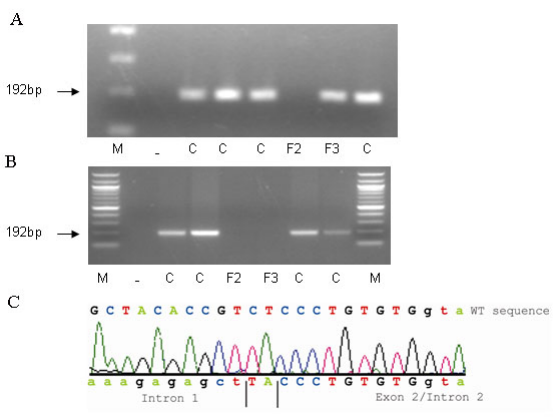
Analysis of the hybrid gene in Family 2. **A** and **B** show agarose gel photographs of PCR products (192 bp) obtained using primer pairs (Table 1) specifically designed to amplify either L exon 2 (**A**) or M exon 2 (**B**). Individual 1.1 of Family 2 is represented by sample F2 on the gels. No amplification products were obtained for sample F2 with either L opsin gene specific primers (**A**) or M opsin gene specific primers (**B**), indicating absence of exon 2 of both L and M opsin genes in Family 2. Sample F3 represents subject 2.3 of Family 3, who has a hybrid gene in which L exon 2 is present (**A**) but M exon 2 (**B**) is absent. These data confirm the absence of both L and M exon 2 in sample F2 (1.1 Family 2) and the presence of L exon 2 and absence of M exon 2 in sample F3 (2.3 Family 3). C denotes male population control sample. Dash (–) denotes a DNA negative control sample, and M indicates a 100 bp DNA ladder. **C** shows sequence of individual 1.1 in Family 2 in which the deletion breakpoint within exon 2 was detected. The subject sequence was compared to the wild type (WT) reference sequence demonstrating that the majority of exon 2 is deleted; intron 1 sequence was joined to terminal exon 2 sequence with 2 bp of intervening sequence.

In a previous study, the underlying molecular cause of BCM in this family was not detected [7]. In the current study, no sequence variants or deletions were identified in the LCR in family member 1.1. Sequence analysis demonstrated the presence of a single gene in the array (Figure 4). Only L exon 1 sequence was detected, and only M exons 3, 4, and 5 were present, indicating the gene is a 5′-L–M-3′ hybrid gene (Figure 3 and Figure 4). Exon 2 failed to amplify in family member 1.1 with a primer pair that coamplifies both L and M exon 2, indicating a possible deletion of exon 2. To test for the absence of an L or M exon 2, we designed specific primer pairs to the L or M gene (Table 1). The primer sequences for L exon 2 and M exon 2 were created to encompass sequence differences between the two genes to specifically amplify either L derived or M derived exon 2 (Table 1). As the primer pair that coamplifies both L and M exon 2 was intronic, it was plausible that family member 1.1 had a polymorphism at the primer annealing site which caused the PCR reactions to fail. However, no L or M exon 2 was detected with L or M specific primers (Table 1 and Figure 5) thereby suggesting that family member 1.1 had a deletion of exon 2. Family members 3.1 and 3.3 were also found to have the same genotype and a lack of L or M exon 2. Long range PCR from L exon 1 to M exon 3 (Table 1) and subsequent direct sequencing resulted in identification of the deletion breakpoint (Figure 5). The deletion spans intron 1 and exon 2 and removes 1,207 bp from the hybrid opsin gene sequence including the splice acceptor for exon 2 and the majority of exon 2 coding sequence (Figure 5). Only 9 bp of exon 2 remained. In summary, BCM in Family 2 is due to nonhomologous recombination resulting in a 5′-L–M-3′ hybrid gene which is rendered inactive by a novel deletion of exon 2 (Figure 3 and Figure 5).

### Family 3 Phenotype

The affected subjects (4.2 and 2.3), age 13 and 65 years respectively, had typical clinical features of BCM and normal myopic fundi. Family member 4.2 underwent electrophysiological assessment, which revealed absent cone responses but normal rod responses. Both had reduced visual acuity with their myopic correction: family member 2.3 had a visual acuity of 6/36 in both eyes and family member 4.2 had a visual acuity of 6/36 right, 6/24 left. Family member 4.2 also underwent detailed psychophysical testing. With the standard and enlarged MR test and HRR plates, good tritan discrimination was detected. He displayed a protan ordering of the D-15, as has been previously described in BCM [24]. On computerized testing, he showed no evidence of color vision on the protan or deutan axis but good discrimination along the tritan axis.

### Family 3 Genotype

**Figure 6:**
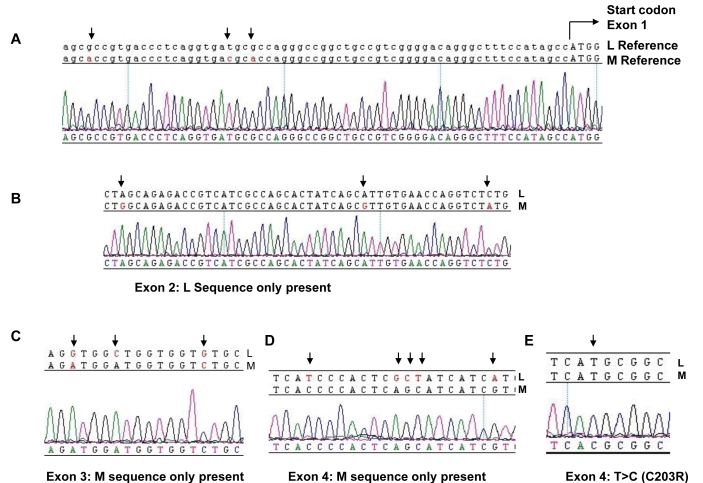
Sequence analysis of L and M opsin genes in Family 3. Reference sequences of the L and M genes are shown on top of the electropherograms generated from an affected male (4.2) in Family 3. Nucleotides marked with an arrow indicate known differences between the L and M genes or a patient mutation. **A** shows sequence 5′ to the start codon (capitals) of exon 1. Note presence of single sequence peaks at positions of known nucleotide variation between L and M opsin sequences (arrows), indicating absence of M exon 1. **B** shows exon 2 sequence with single peaks representing the presence of L nucleotides but absence of M nucleotides at sites of known variation between L and M opsin genes. **C** shows exon 3 sequence in which M exon 3 only is present. **D** shows exon 4 sequence in which only M exon 4 sequence is present. **E** shows exon 4 sequence with a T>C nucleotide substitution (arrow) resulting in C203R missense mutation.

No sequence variants or deletions were identified in the LCR. Sequence analysis demonstrated the presence of L exon 1 and 2, and M exon 3, 4, and 5 only (Figure 6) as a single hybrid 5′-L–M-3′ gene (Figure 3). The sequence results, which indicated the presence of only L exon 2 in this family, were confirmed by specific exon 2 amplification as described for Family 2. Figure 5 shows that family member 2.3 (sample F3) had L exon 2 (Figure 5A) but no M exon 2 (Figure 5B). Sequence analysis of exon 4 revealed that this hybrid opsin gene carries a T>C nucleotide substitution, encoding a C203R missense mutation (Figure 6E and Figure 2D). Family member 4.2 was also found to have the same genotype. In contrast to Family 1, the hybrid opsin gene in this family consisted of L exons 1 and 2 joined to M exons 3 to 6, indicating that nonhomologous intragenic recombination took place between L exon 2 and M exon 3 (Figure 3). In summary, BCM in Family 3 is also due to a two-step mutational mechanism where nonhomologous recombination has resulted in a 5′-L–M-3′ hybrid gene that is rendered inactive by a C203R missense mutation in exon 4 (Figure 3, Figure 5, and Figure 6).

## Discussion

We have identified the molecular genetic basis of BCM in each of the three families described, with one family harboring a novel disease-causing genotype. In all cases genetic rearrangements of the L and M opsin array were found that would result in a lack of functional L and M pigments, and thus inactivate the corresponding cones. The genetic mechanism leading to BCM in two of these families requires a two-step pathway of nonhomologous recombination followed by an intragenic mutation. In family 2 the single hybrid gene lacking exon 2 is likely to have occurred by a single nonhomologous recombination event. Two out of the 3 families (Families 2 and 3) had a single hybrid gene in the array that was inactivated either by a point mutation (C203R in Family 3) or by a deletion (exon 2 in Family 2). In the remaining family (Family 1), the array is probably composed of a hybrid gene adjacent to a second gene (either an M gene or a second hybrid), both of which are nonfunctional. Inactivation of the genes in this array is due either to the presence of a C203R mutation in the first two genes of the array or to a C203R substitution in the first and the deletion of several exons in the second.

The C203R mutation has been identified previously in several molecular genetic studies of BCM [7,9,15]. The C203R substitution destabilizes the L and M opsin proteins by disrupting a highly conserved disulphide bond which plays a crucial role in the tertiary structure of both rod and cone opsins [23]. We identified this inactivating mutation in two out of the three families, thereby providing further evidence that C203R is the most frequent point mutation in BCM. Four British families are now known to harbor the C203R mutation. There are no reports of LCR deletions in this population [7, and this article]. As more families are diagnosed clinically and the molecular basis of disease is defined, it will be interesting to see if this reflects a population difference in mutational mechanism, compared with, for example, the United States patient population, in whom LCR deletions have been found in up to 40% of cases [9,15-17].

The genotype identified in Family 2 of a single hybrid gene inactivated by a lack of exon 2 represents a novel mutation resulting in BCM. The only previously reported similar class of mutation was in a Danish family in whom exon 4 of a single L opsin gene had been deleted [18]. This therefore represents the second example of an uncommon category of BCM mutations that involves an intragenic exonic deletion in a single gene in the array. Interestingly, the exon 4 deletion in the Danish family is predicted to result in a frameshift mutation by splicing exon 3 to exon 5, which would alter the reading frame of the L cone opsin gene and generate a truncated protein with a premature stop at codon 266 [18]. It is possible that this frameshift mutation would be subject to nonsense mediated mRNA decay. In direct contrast, the novel exon 2 deletion we detected in Family 2 would not result in a shift of the reading frame. Instead, it is predicted that exon 1 is spliced in-frame to exons 3–6 and a protein product may be generated. The protein would be nonfunctional as exon 2 codes for a major part of the opsin. The mutant protein product would lack part of the N-terminal extracellular domain, transmembrane helix I, cytoplasmic loop I, transmembrane domain II, extracellular loop I and part of transmembrane helix III. We hypothesize that this mutant partial “opsin” protein could accumulate in L and M cone photoreceptors that express this hybrid gene product over time, leading to gradual cone cell loss and a slowly progressive phenotype as observed in Family 2.

In addition to Family 2 there are a small number of other families with BCM in which slow disease progression has been documented [7,9,17,25]. Molecular genetic studies in three of the families have shown an LCR deletion in two families [9,17] and a single L–M hybrid with an inactivating C203R mutation in the third family [7]. Progression was not reported in the Danish family in whom exon 4 of an isolated L opsin gene had been deleted [18]. Our findings in Family 2 provide further evidence of intragenic heterogeneity even among families with a slowly progressive BCM phenotype. It is difficult to postulate a molecular mechanism underlying disease progression in the families with either LCR deletions or single inactive hybrid genes. These genotypes are the most commonly identified mutants in BCM, yet most of the affected individuals in these families have a stationary disorder. It is possible, however, that unidentified modifying genes or environmental factors may account for this observed phenotypic heterogeneity. It is also probable that if more detailed phenotyping, including psychophysical testing, were employed over time in more patients with BCM, progression might be detected more commonly than expected.
